# Radiological characteristics of pulmonary cryptococcosis in HIV-infected patients

**DOI:** 10.1371/journal.pone.0173858

**Published:** 2017-03-16

**Authors:** Zhiliang Hu, Jun Chen, Juan Wang, Qingfang Xiong, Yandan Zhong, Yongfeng Yang, Chuanjun Xu, Hongxia Wei

**Affiliations:** 1 Department of Infectious Disease, the Second Affiliated Hospital of Medical School of the Southeast University, Nanjing, Jiangsu, China; 2 Department of Infectious Diseases, Shanghai Public Health Clinical Center, Fudan University, Shanghai, China; 3 Department of Pathology, Nanjing Brain Hospital, Nanjing Medical University, Nanjing, Jiangsu, China; 4 Department of radiology, the Second Affiliated Hospital of Medical School of the Southeast University, Nanjing, Jiangsu, China; ARGENTINA

## Abstract

**Background:**

Current understanding of human immunodeficiency virus (HIV)-associated pulmonary cryptococcosis (PC) is largely based on studies performed about 2 decades ago which reported that the most common findings on chest radiograph were diffuse interstitial infiltrates. Few studies are available regarding the computed tomography (CT) findings. The aim of this study was to characterize chest CT features of HIV-associated PC.

**Methods:**

HIV patients with cryptococccal infection and pulmonary abnormalities on Chest CT between September 2010 and May 2016 in the Second Affiliated Hospital of the Southeast University were retrospectively analyzed. Confirmed cases of tumors, mycobacterial infections and other fungal infections were excluded from the analysis.

**Results:**

60 cases were identified. The median CD4 T-cell counts were 20 cells/μL (range, 0–205 cells/μL). Chest CT scans demonstrated nodular lesions in 93.3% of the studied patients. Those nodular lesions were usually cavitated and solitary nodule was the most common form. Pleural effusions and pneumonic infiltrates occurred in 11.6% and 31.7% of the cases respectively. Those lesions were usually had co-existing nodular lesions. Etiological analysis suggested that 76.8% of the nodular lesions could have a relationship with PC that 12.5% of the nodular lesions were “laboratory-confirmed” cases, 48.2% were “clinically confirmed” cases and 16.1% were “clinically probable” cases. 85.7% of the pleural effusions could be “clinically confirmed” cases of PC. At least, 38.5% of the diffuse pneumonic infiltrates may be clinically attributed to pneumocystis pneumonia.

**Conclusions:**

This study suggested that pulmonary nodules but not diffuse pneumonia are the most common radiological characteristics of HIV-associated PC. HIV-infected patients with pulmonary nodules on Chest CT should particularly be screened for cryptococcal infection.

## Introduction

In human immunodeficiency virus (HIV)-negative patients, pulmonary cryptococcosis (PC) has been extensively studied and nodular masses are the most common radiological manifestations [1–4]. However, PC in HIV-infected is less well defined. Knowledge of HIV-associated PC are largely based on studies performed about 2 decades ago which reported that the most common findings on chest radiograph were diffuse interstitial infiltrates [5–7]. In those HIV-infected immunocompromised patients, PC was thought to be an important contributor to fetal respiratory failure [5]. Although computed tomography (CT) has been widely applied in evaluating pulmonary lesions, few studies on the CT findings of HIV-associated PC are available. In our previous study, we evaluated 11 HIV-infected patients with pulmonary abnormalities on chest CT scan and confirmed cryptococcal diseases outside of the lung. Interestingly, 9 patients had solitary pulmonary nodules that could be clinically related to cryptococcal infection [8]. This finding is inconsistent with the dominant perception of HIV-associated PC and necessitates further investigation. Here, we extend our previous study to expand the understanding of CT findings of HIV-associated PC.

## Patients and methods

### Patients

HIV-infected patients with crytococccal infection diagnosed by positive cryptococcal antigen test and/or isolation of *Cryptococcus* spp., between September 2010 and May 2016 in the Second Affiliated Hospital of the Southeast University, were retrospectively analyzed. Those with pulmonary abnormalities were further evaluated for the possibility of pulmonary cryptococcosis. Confirmed cases of tumors, mycobacterial infections and other fungal infections were excluded from the analysis. The medical records, such as age, sex, antiretroviral therapy, clinical features, laboratory tests, chest CT scan imaging results, treatment and outcome, were collected. This retrospective study was approved by the Ethics Committee of the Second Affiliated Hospital of the Southeast University. The data were analyzed and presented anonymously.

### Radiological assessment

The chest CT scan findings were mainly classified into three patterns, which were the nodular lesion with clear boundary, the pneumonic infiltrates with ill-defined margin, and pleural effusions, as described by Zhang et al with modification[2]. Based on the number of the nodule, nodular lesions were further subdivided into single nodular (solitary) and multi-nodular. Cavitation was also recorded. The pneumonic infiltrateswere subdivided into focal, multi-focal and diffuse pneumonic infiltrates. The special pneumonic infiltrates, defined as diffuse ground-glass opacities on CT scans, were also recorded.

### Diagnosis of PC

If pulmonary lesion responded to anti-cryptococcal therapy alone, the case was defined as “clinically confirmed” PC. If additional drugs were also used to achieve an improvement, PC was considered “clinically probable”. PC was considered “laboratory confirmed” if cryptococcal elements were revealed by lung biopsy or sputum culture from a “clinically confirmed” PC patient grew *Cryptococcus*spp.. PC was considered “unlikely” if pulmonary lesions improved without an anti-cryptococcal treatment. The special lesions of diffuse ground-glass opacitieswere attributed to pneumocystis pneumonia (PCP) if lesions resolved with anti-PCP therapy alone.

## Results

### Identification of cases

From September 2010 to May 2016, 80 HIV-infected patients were confirmed to have cryptococcal infection in the Second Affiliated Hospital of the Southeast University, of whom 66 cases have pulmonary abnormalities on chest CT scan. After excluded 4 cases of pulmonary tuberculosis and 2 cases of Kaposi’s sarcoma, the remaining 60 cases with pulmonary abnormalities were further evaluated for the possibility of PC(S1 Table). Of these 60 cases, 88.3% (53/60) were men and 76.7% (46/60) had cryptococcal meningitis (CM). 44 patients with cryptococcal meningitis were treated with amphotericin B-based combination therapy followed by fluconazole treatment as recommended by clinical practice guidelines [9]. Of those 44 patients treated with amphotericin B, 32 patients had blood culture results available that 56.3% (18/32) of the blood cultures grew *Cryptococcus* spp. Patients without CM were treated with fluconazole alone. The median age was 38 years (range, 12-71years). The median CD4 T-cell counts were 20 cells/μL (range, 0–205 cells/μL). Six patients had initiated anti-retroviral therapy for a median of 3 months (range, 2 weeks to 2 years), of whom two developed virological failure.

### Findings on chest CT scan

**Fig 1:**
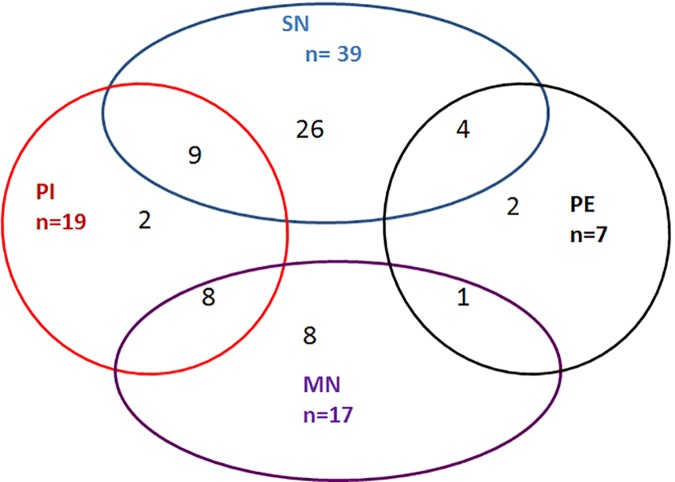
Chest CT findings of HIV-infected patients with cryptococcal infection and pulmonary abnormalities. SN, solitary nodule; MN, multiple nodules; PE, pleural effusions; PI, pneumonic infiltrates.

Chest CT scans demonstrated nodular lesions in 93.3% (56/60) of the studied patients (Fig 1), and solitary nodule accounted for the majority of the cases (69.6%, 39/56). These nodular lesions were usually cavitated with a cavitation rate of 78.6% (44/56). Pleural effusions occurred in 11.6% (7/60) of the studied patients, and 71.4% (5/7) of the patients with pleural effusions had co-existing nodular lesions. Pneumonic infiltrates occurred in 31.7% (19/60) of the patients; of which, 68.4% (13/19) were diffuse pneumonic infiltrates (all diffuse ground-glass opacities), 21.1% (4/19) were focal pneumonic infiltrates and 10.5% (2/19) were multi-focal pneumonic infiltrates. Of these 19 patients with pneumonic infiltrates, 89.5% (17/19) had co-existing nodular lesions.

### Etiologies of pulmonary lesions

**Fig 2:**
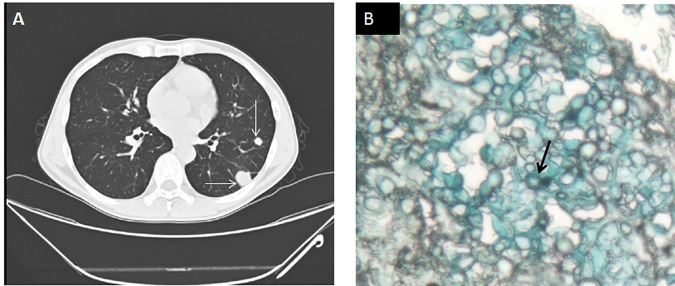
A representative case of laboratory-confirmed pulmonary cryptococcosis. A 49-years-old man presented to hospital with Lymphadenopathy for 3 months and fever for 1 month. He had HIV infection with a CD4 count of 108 cells/μL. Serum cryptococcal antigen titer was 1:40. Serum galactomannan assay was negative. Chest CT scan demonstrated multiple pulmonary nodules (A, arrow). Lung biopsy showed encapsulated yeast-like fungal cells consistent with cryptococcal infection(arrows in B, alcian blue stain).

76.8% (43/56) of the nodular lesions could be demonstrated a relationship with PC that 12.5% (7 /56) of the nodular lesions were “laboratory-confirmed” cases, 48.2% (27/56) were “clinically confirmed” cases and 16.1% (9/56) were “clinically probable” cases (Table 1). Of the 7 “laboratory-confirmed” cases of PC with nodular lesions, 5 were confirmed by lung needle biopsies that revealed cryptococcal elements (Fig 2, S1 File) and culture of the biopsy specimens grew *Cryptococcus* spp.; the other 2 “laboratory-confirmed” cases of PC fulfill the criteria of “clinically confirmed” cases and culture of the sputum grew *Cryptococcus*spp. (S1 File). All the above mentioned 9 “clinically probable” cases of PC also had pneumonic infiltrates that anti-cryptococcal drugs and other antibiotics were used to achieve an improvement of pulmonary lesions(S1 File). Finally, it was difficult to deduce the etiologies of the nodular lesions in 23.2% (13/56) of the patients because follow-up chest CT scans to define clinical responses were unavailable(S1 File).

**Table 1:** Etiologies of pulmonary lesions.

			Etiologies of the pulmonary lesions^a^					
Pulmonary lesions	Case No.	Cases with respiratory Symptoms	PC Laboratory confirmed	PC clinically confirmed	PC clinically probable	PC Unlikely	Undefined^b^	Multiple Infection^c^
SN	26	7	3	14	-	-	9	-
SN+PI	9	9	1	-	4	-	2	2
SN+PE	4	3	-	3	-	-	1	-
MN	8	4	3	5	-	-	-	-
MN+PI	8	7	-	-	5	-	1	2
MN+PE	1	0	-	1	-	-	-	-
PI	2	0	-	-	1	1^d^	-	-
PE	2	1	-	2	-	-	-	-
Total	60	31	7	25	10	1	13	4

PC, pulmonary cryptococcosis; SN, solitary nodule; PI, pneumonic infiltrates; PE, pleural effusions; MN, multiple nodules.

^a^: Lung needle biopsies were performed in five patients which confirmed PC. Sputum cultures were available in 33 patients, of whom 2 grew *Cryptococcus* spp., 10 grew Candida albicans thought to be related to oral candidiasis, and the remaining 21 showed negative results.

^b^: Etiologies of pulmonary lesions were undefined as clinical data were insufficient.

^c^: All these 4 patients had mixed pulmonary lesions. By thoroughly analyzing the clinical data, nodular lesions were considered “clinically confirmed” cases of PC. The diffuse pneumonic infiltrates, specifically the diffuse ground-glass opacities, were attributed to pneumocystis pneumonia

^d^: Before admitted to our hospital, this patient had severe respiratory symptoms and chest CT scan showed diffuse ground-glass opacities. After treated with empirical co-trimoxazole for pneumocystis pneumonia, respiratory symptoms gradually improved. When he presented to our hospital with neurological symptoms, he had received about 3 weeks of co-trimoxazole and did not complain any respiratory symptoms. Chest CT scan showed residual ground-glass opacities thought to be related to previous pneumocystis pneumonia.

**Fig 3:**
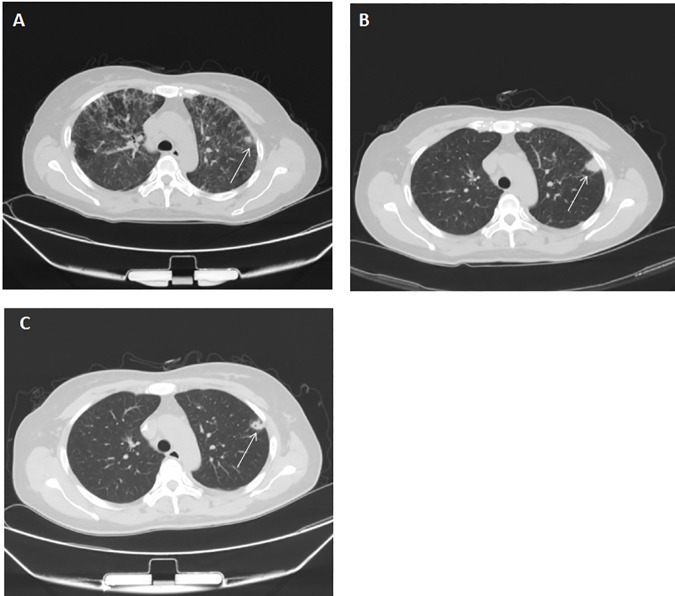
A representative case of clinical deduction of pulmonary cryptococcosis. A 47-year-old woman presented to hospital with fever, cough and shortness of breath. She was confirmed of HIV infection with a CD4 count of 60 cells/μL. The serum cryptococcal antigen titer was 1:16. Lumbar puncture was performed and there was no evidence of cryptococcal meningitis. A chest CT scan showed diffuse ground-glass opacities (DGO). Also on the Chest CT, there was a small nodule (A, arrow). She was given empirical treatment with co-trimoxazole (1440 mg, every eight hours) for pneumocystis pneumonia, and methylprednisolone as an adjunctive therapy. A chest CT scan after 3 weeks showed nearly complete resolution of the DGO; however, the nodular lesion progressed(B, arrow). The patient then received secondary prophylaxis for pneumocystis pneumonia with co-trimoxazole(960 mg/d) and treated with fluconazole (400mg/d). A follow-up CT scan after seventeen days of fluconazole treatment showed partial resolution of the nodule (C, arrow). Taken together, the nodule lesion was attributed to pulmonary cryptpcoccosis, while the DGO were related to pneumocystis pneumonia.

85.7% (6/7) of the pleural effusions were “clinically confirmed” cases of PC, and the remaining one was undefined due to lack of follow-up chest CT scan. For the 19 cases of pneumonic infiltrates, one with solitary cavitary nodule and multi-focal pneumonia fulfill the criteria of “clinically confirmed” case and culture of the sputum grew *Cryptococcus* spp., therefore was considered “laboratory-confirmed” PC. Of the 13 cases of diffuse pneumonic infiltrates, 53.8% (7/13) were "clinical probable" cases of PC as additional drugs, aside from anti-cryptococcal drugs, were used to achieve pulmonary improvement (those 7 cases also received anti-PCP therapy therefore PCP could not be excluded); 38.5% (5/13) were unlikely to related to PC (Fig 3, a representative case of clinically deducing the etiology of pulmonary lesion) but were perhaps clinically attributed to PCP. Etiology of diffuse pneumonic infiltrates was undefined in 1 case due to lack of follow-up chest CT scan.

## Discussion

In this study, 82.5% (66/80) of the HIV-infected patients with confirmed cryptococcal infection had pulmonary lesions. On one hand, those lesions could be related to cryptococcal infection, but on the other hand those lesions may be caused by other pathogens or tumors. Due to immunodeficiency, mixed infection should not beunderestimated, especially in patients with multiple forms of pulmonary lesions. The initial identification of possible cases of PC excluded 4 cases of pulmonary tuberculosis and 2 cases of Kaposi’s sarcoma. Intriguingly, 56 out of the 60 remaining cases showed pulmonary nodules which usually cavitated and solitary nodule was the most common form. Based on the available patients' data, at least 76.8% (43/56) of the nodular lesions could be demonstrated a relationship with PC. Those findings were closely consistent with our previous observation which suggested that solitary cavitary pulmonary nodule may be a common CT finding in HIV-associated PC[8]. Nevertheless, pulmonary nodular lesions with or without cavitation, although reported in other studies, were not recognized as common manifestations of HIV-associated PC [6, 7, 10]. More recently, Lin et al reported that PC contributed to about 17% of the cavitary pulmonary lesions in HIV-infected patients [11].

The high frequency of diffuse interstitial infiltrates, attribute to HIV-associated PC in other studies [6, 7], was not seen in our study. Diffuse pneumonic infiltrates, specifically diffuse ground-glass opacities, were only present in 21.7% of (13/60) the patients and almost always co-existed with pulmonary nodules. Of note, at least some of those diffuse pneumonic infiltrates may be related to PCP (Table 1, Fig 3). It is therefore better to include anti-cryptococcal therapy as well as anti-PCP drugs in empirical treatment for patients with confirmed cryptococcal infection (for example CM) and diffuse pneumonic infiltrates. Aside from PCP, it is also necessary to exclude other causes of diffuse pneumonic infiltrates in HIV-infected immunocompromised persons, such as cytomegalovirus pneumonia and mycobacteriosis[12, 13]. This is important as diffuse pneumonic infiltrates, unlike nodular lesion, would result in severe respiratory symptoms, and may quickly progress to respiratory failure if left untreated.

Although pleural effusions were less frequently seen in our study, the presence of pleural effusions was suggestive of PC in our patients. Of note, pleural effusions could be caused by multiple common conditions in HIV-infected patients, such as tuberculosis and Kaposi sarcoma [13, 14]. Extensive laboratory evaluation of pleural effusions was required torule out those comorbidities, especially when pleural effusions did not respond to anti-cryptococcal therapy.

As discussed above, cryptococcal infection seems likely to cause localized pulmonary lesions in HIV-infected patients similar with that in HIV-negative patients [1–4], although cavitation of the nodules seems to be more common in HIV-infected patients. The imaging modality of Chest CT scan used in our study is more sensitive to reveal pulmonary lesions [15]. As shown in Fig 3, the small nodular lesion was surrounded with diffuse pneumonic infiltrates which may be very difficult to be revealed by chest radiograph. It is possible that nodular lesions were underestimated in former studies of HIV-associated PC[5–7]. Moreover, as shown in our study and others’ case report[10], pulmonary nodules without pneumonic infiltrates or pleural effusions did not often cause respiratory symptoms, while former studies on HIV-associated PC generally included patients with respiratory symptoms [5–7]. All the above mentioned factors may lead to the low prevalence of pulmonary nodules in HIV-associated PC in other studies. Nevertheless, as our study was conducted in Chinese population, different host-pathogen interactions may exist and contribute to the discrepancy.

There are limitations to our study. Only a minority of the cases of PC were laboratory-confirmed, and most cases were diagnosed by clinical deduction. Response of pulmonary lesions to anti-cryptococcal treatment is suggestive of PC, however other fungal infections, such as aspergillosis, penicilliosismarneffei and histoplasmosis that could cause similar lesions [16–18], should also be excluded. Nevertheless, the clinically confirmed cases of PC were less likely to be associated with aspergillosis, penicilliosismarneffei or histoplasmosis, as anti-cryptococcal treatments used in our study were not optimal therapies for the above mentioned diseases[19]. Also, penicilliosis was unlikely considered because our patients were not from an endemic area and did not have skin lesions or blood culture results consistent with penicilliosis. Aside from fungal infections, pulmonary nodules could also be caused by many other reasons, such as mycobacteria infection and malignancy [11, 13, 20–22], and those etiologies should be included in the differential diagnosis.

In conclusion, our study suggested that pulmonary nodules but not diffuse pneumonia are the most common radiological characteristics of HIV-associated PC. HIV-infected patients with pulmonary nodules on chest CT should particularly be screened for cryptococcal infection.

## Supporting information

S1 Table Patients’ data. CMV, cytomegalovirus; CT, computed tomography; PC, pulmonary cryptococcosis; PCP, pneumocystis pneumonia. a: The lower limit of detection for CMV was 500 copies/mL. b: All the patients with CD4 cell counts less than 200 cells/μL received prophylaxis therapies for pneumocystis pneumonia. Those treatments were not shown in the category of other anti-infective therapies.
(XLSX)

S1 File Representative cases of laboratory-confirmed, clinically confirmed, clinically probable pulmonary cryptococcosis and undefined pulmonary lesions. Cases 1–6 were laboratory-confirmed pulmonary cryptococcosis. Cases 7 and 8 were clinically confirmed pulmonary cryptococcosis. Cases 9 and 10 were clinically probable pulmonary cryptococcosis. Etiologies of the pulmonary lesions of Cases 11 and 12 were undefined.
(PPTX)
