# Supplementary material for: Radiological characteristics of pulmonary cryptococcosis in HIV-infected patients
Source: PLoS One. 2017 Mar 16;12(3):e0173858. doi: 10.1371/journal.pone.0173858 (PMC5354418; doi:10.1371/journal.pone.0173858)
Supplement: S1 File — Cases 1–6 were laboratory-confirmed pulmonary cryptococcosis. Cases 7 and 8 were clinically confirmed pulmonary cryptococcosis. Cases 9 and 10 were clinically probable pulmonary cryptococcosis. Etiologies of the pulmonary lesions of Cases 11 and 12 were undefined. (PPTX) [file pone.0173858.s002.pptx]

## Slide 1
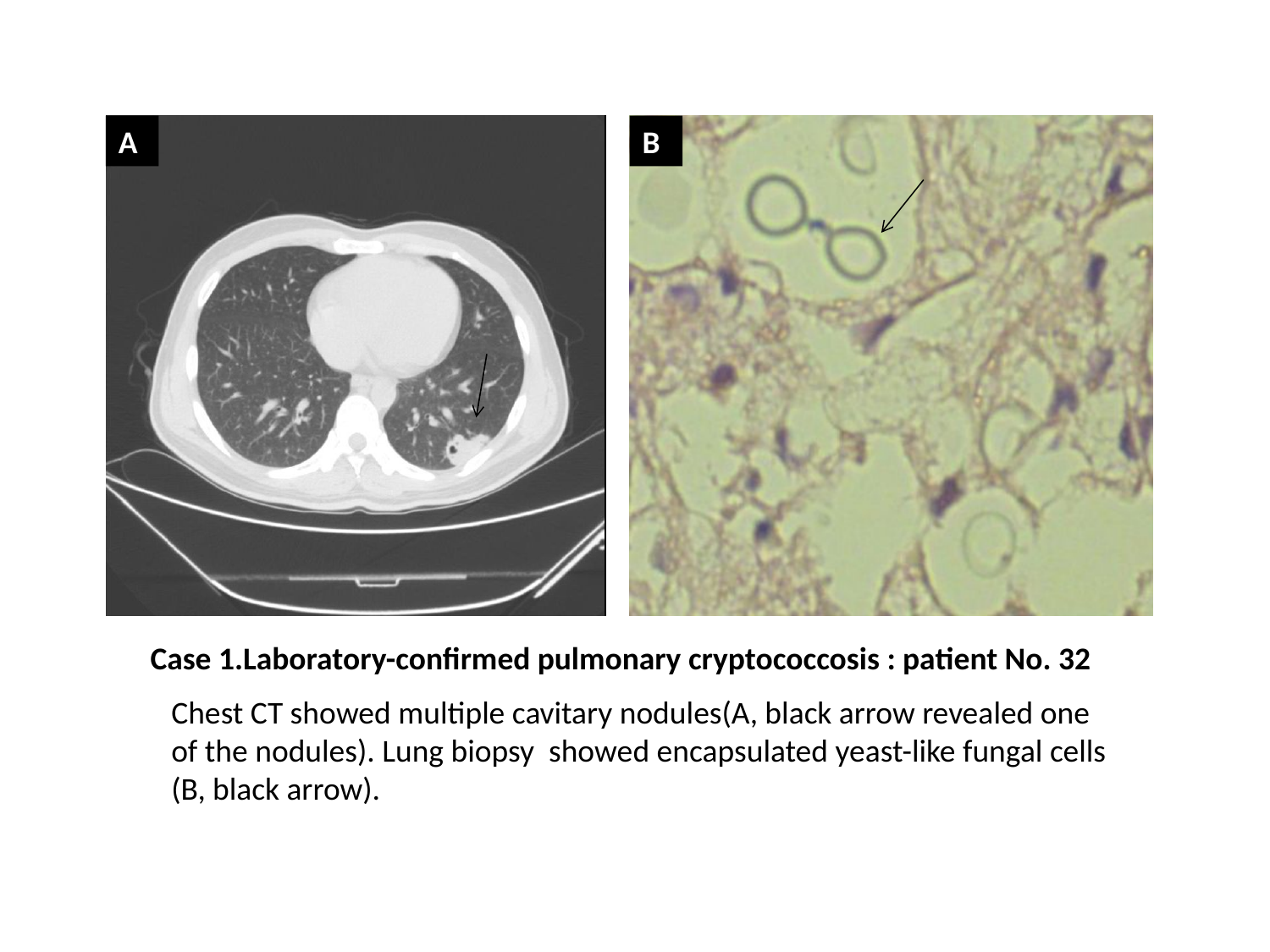

A
B
Case 1.Laboratory-confirmed pulmonary cryptococcosis : patient No. 32
Chest CT showed multiple cavitary nodules(A, black arrow revealed one of the nodules). Lung biopsy showed encapsulated yeast-like fungal cells (B, black arrow).

## Slide 2
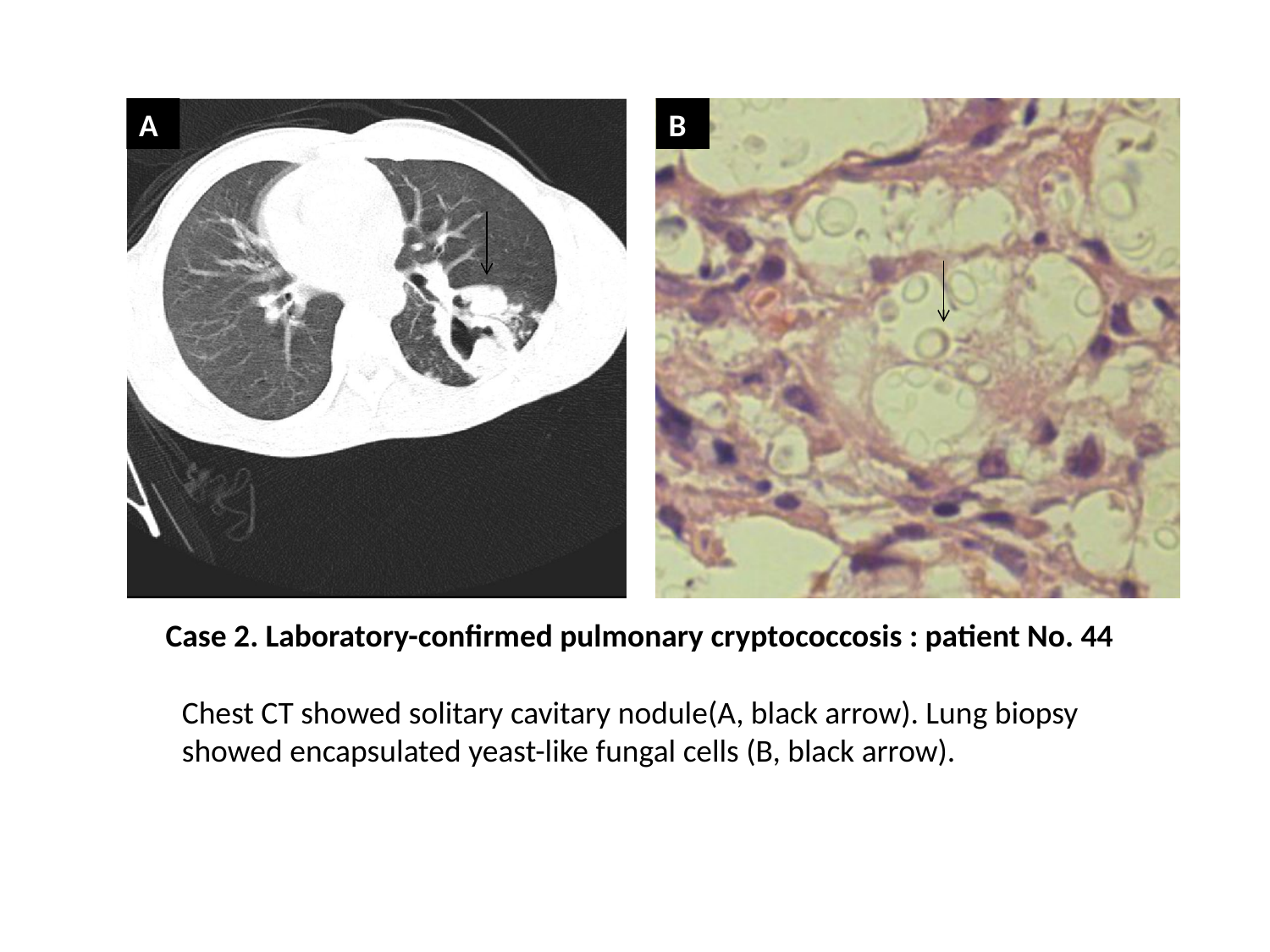

A
B
Case 2. Laboratory-confirmed pulmonary cryptococcosis : patient No. 44
Chest CT showed solitary cavitary nodule(A, black arrow). Lung biopsy showed encapsulated yeast-like fungal cells (B, black arrow).

## Slide 3
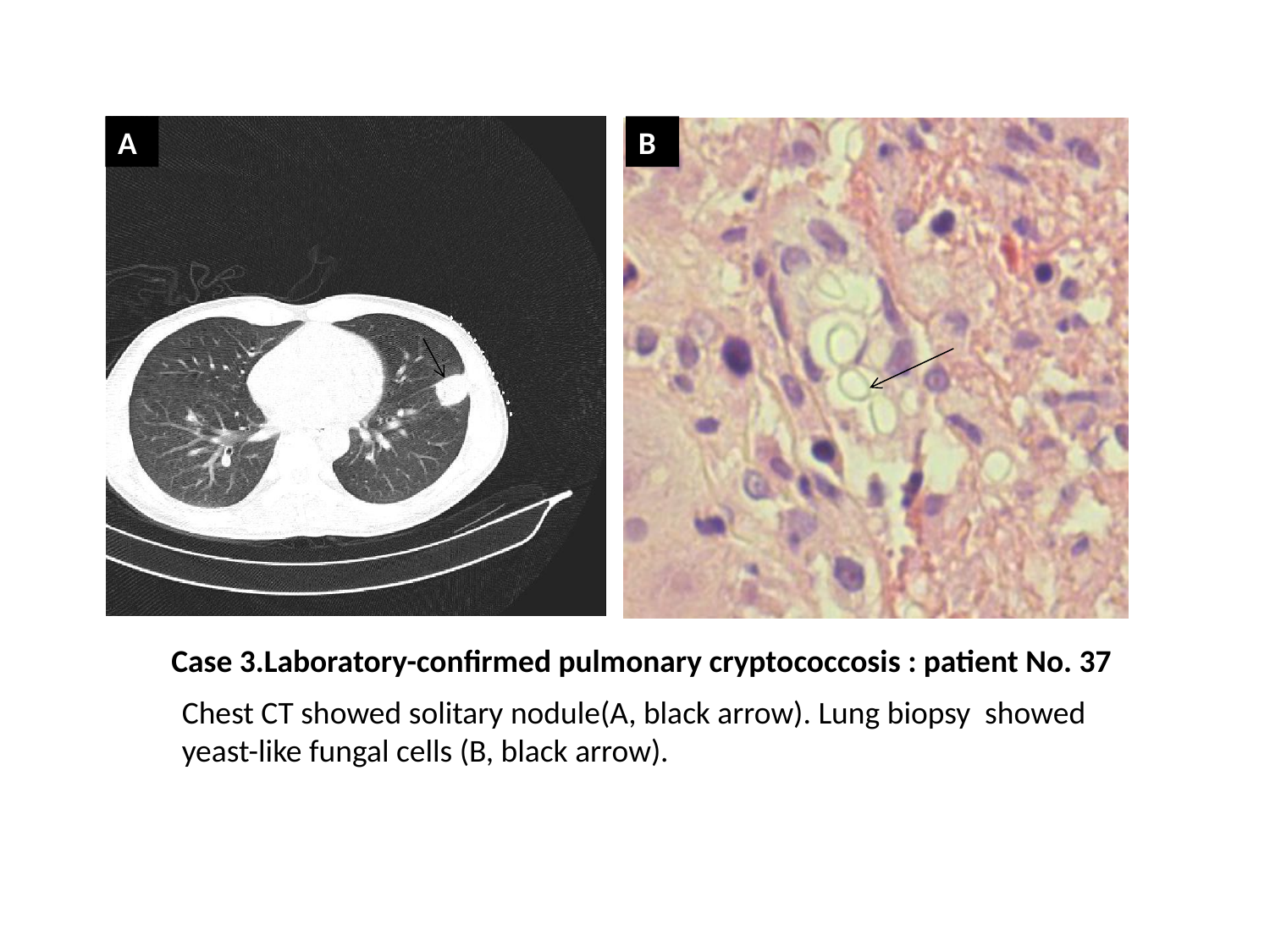

A
B
Case 3.Laboratory-confirmed pulmonary cryptococcosis : patient No. 37
Chest CT showed solitary nodule(A, black arrow). Lung biopsy showed yeast-like fungal cells (B, black arrow).

## Slide 4
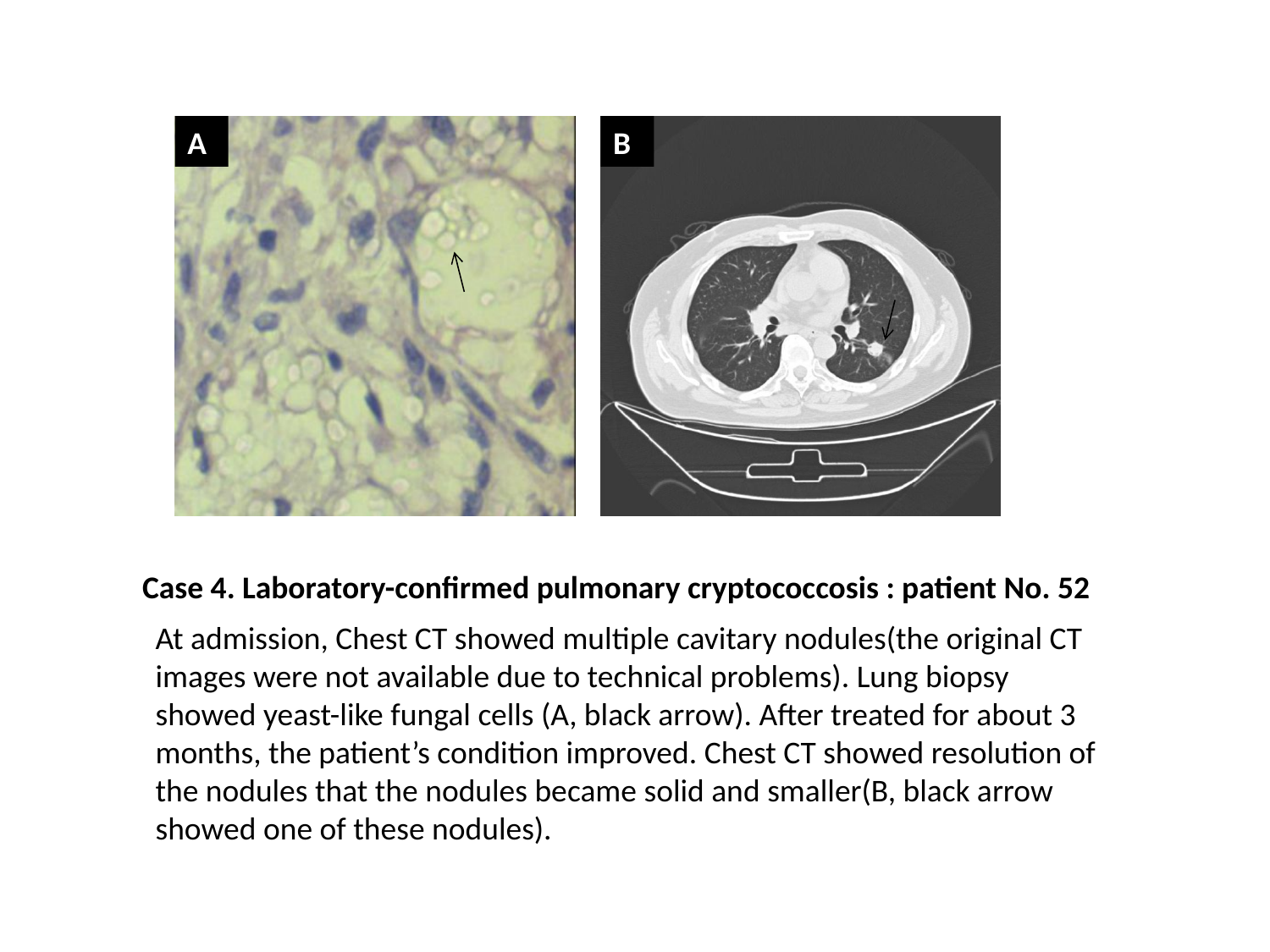

A
B
Case 4. Laboratory-confirmed pulmonary cryptococcosis : patient No. 52
At admission, Chest CT showed multiple cavitary nodules(the original CT images were not available due to technical problems). Lung biopsy showed yeast-like fungal cells (A, black arrow). After treated for about 3 months, the patient’s condition improved. Chest CT showed resolution of the nodules that the nodules became solid and smaller(B, black arrow showed one of these nodules).

## Slide 5
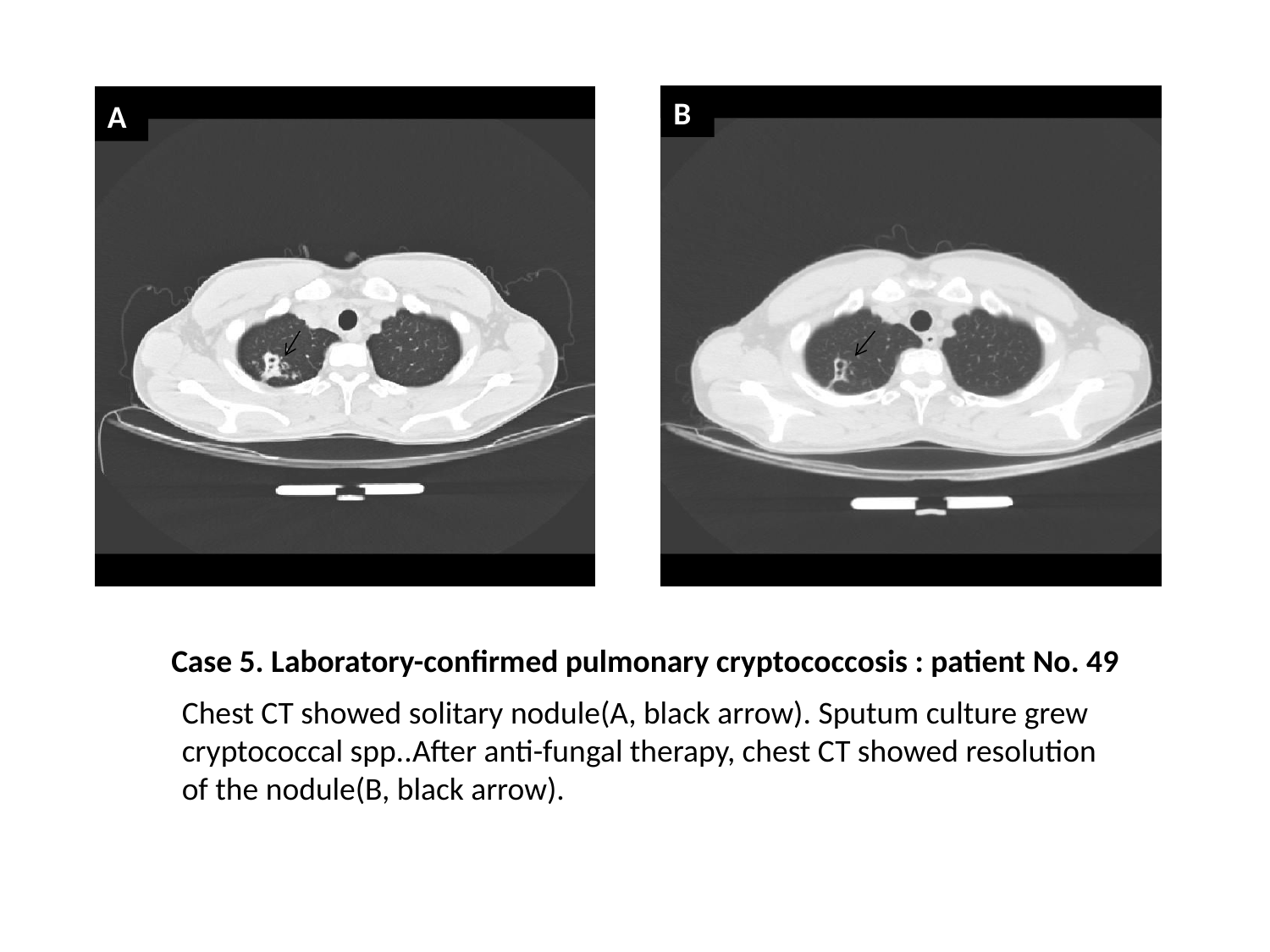

B
A
Case 5. Laboratory-confirmed pulmonary cryptococcosis : patient No. 49
Chest CT showed solitary nodule(A, black arrow). Sputum culture grew cryptococcal spp..After anti-fungal therapy, chest CT showed resolution of the nodule(B, black arrow).

## Slide 6
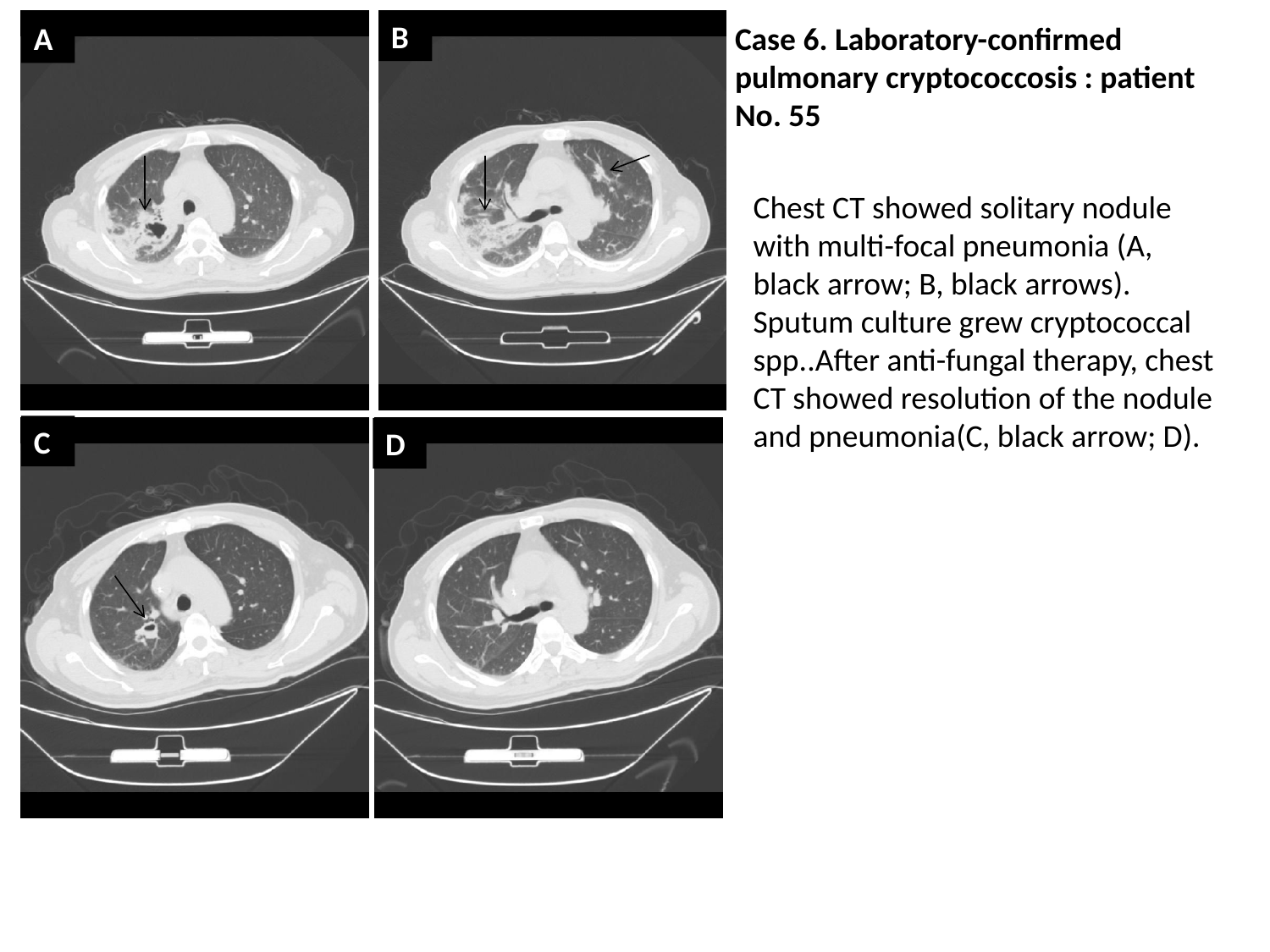

B
A
Case 6. Laboratory-confirmed pulmonary cryptococcosis : patient No. 55
Chest CT showed solitary nodule with multi-focal pneumonia (A, black arrow; B, black arrows). Sputum culture grew cryptococcal spp..After anti-fungal therapy, chest CT showed resolution of the nodule and pneumonia(C, black arrow; D).
C
D

## Slide 7
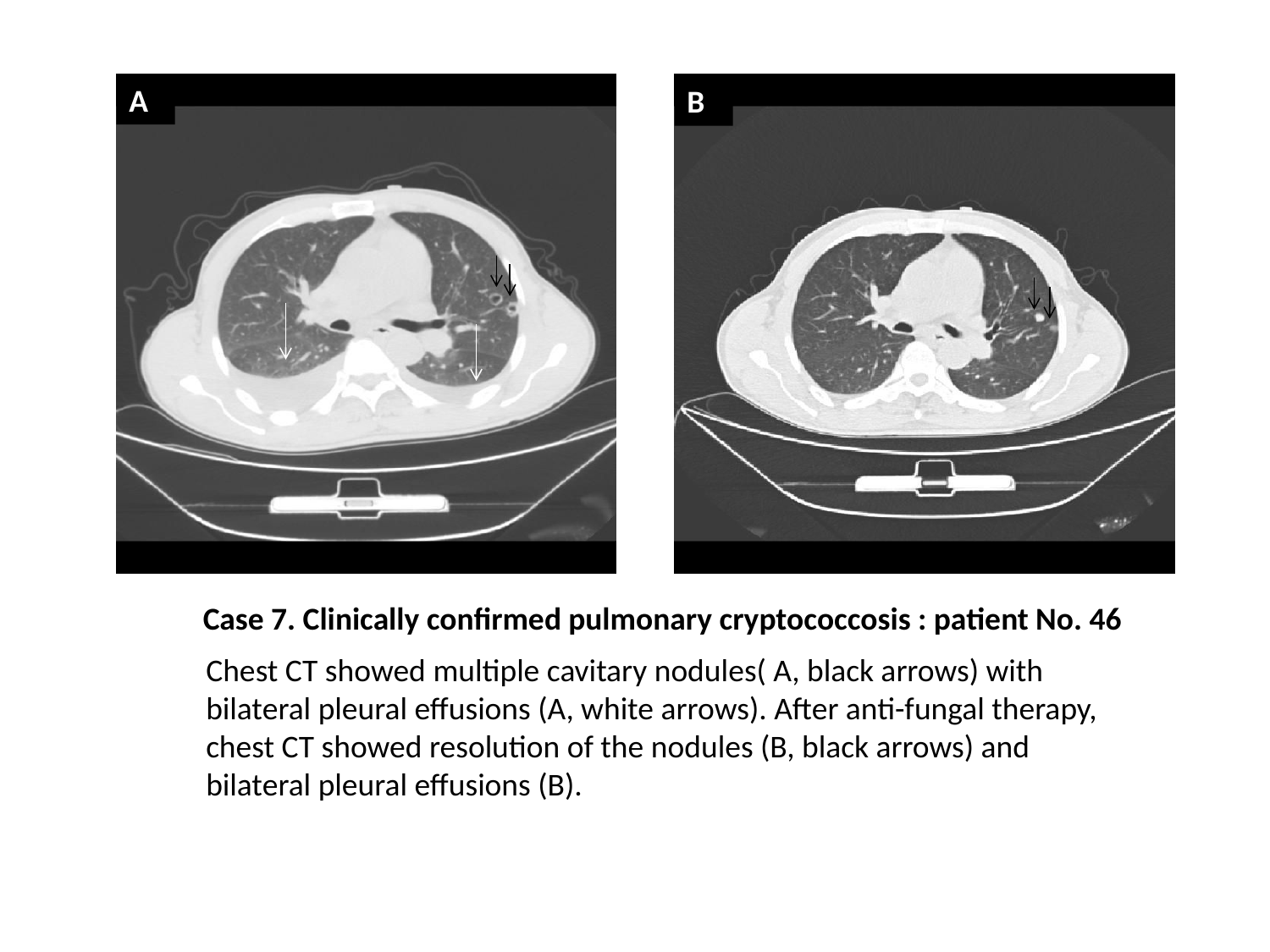

A
B
Case 7. Clinically confirmed pulmonary cryptococcosis : patient No. 46
Chest CT showed multiple cavitary nodules( A, black arrows) with bilateral pleural effusions (A, white arrows). After anti-fungal therapy, chest CT showed resolution of the nodules (B, black arrows) and bilateral pleural effusions (B).

## Slide 8
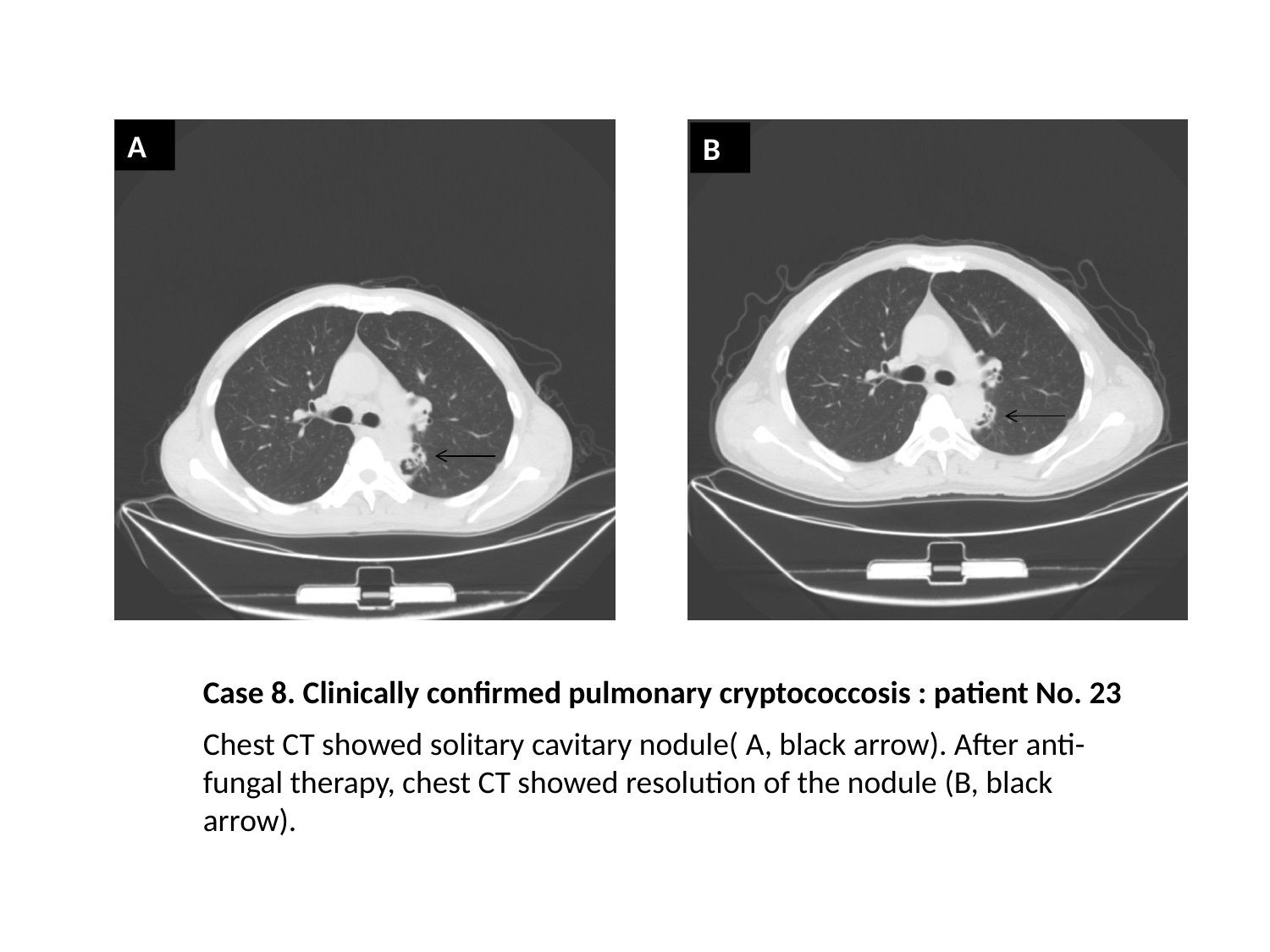

A
B
Case 8. Clinically confirmed pulmonary cryptococcosis : patient No. 23
Chest CT showed solitary cavitary nodule( A, black arrow). After anti-fungal therapy, chest CT showed resolution of the nodule (B, black arrow).

## Slide 9
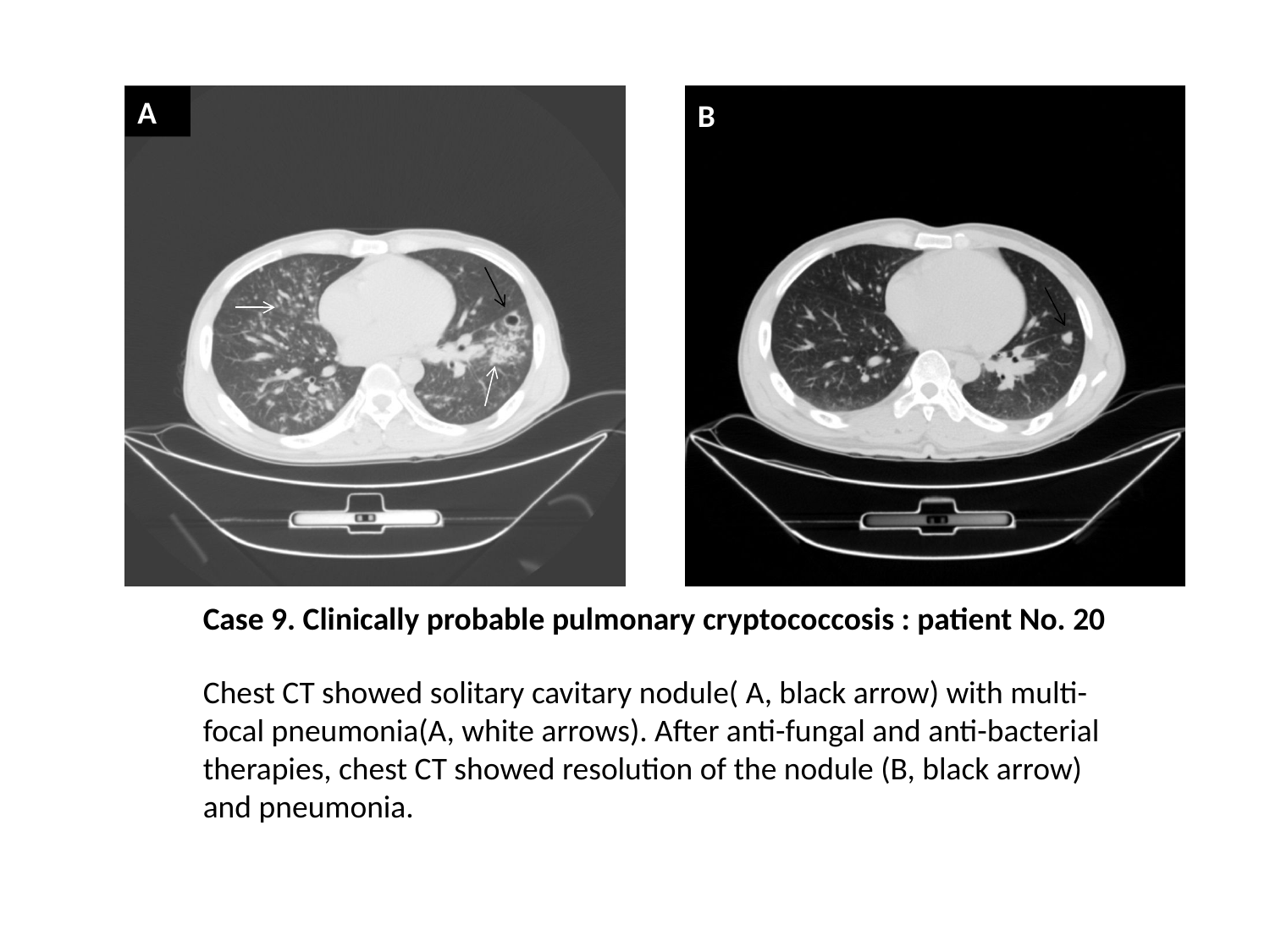

A
B
Case 9. Clinically probable pulmonary cryptococcosis : patient No. 20
Chest CT showed solitary cavitary nodule( A, black arrow) with multi-focal pneumonia(A, white arrows). After anti-fungal and anti-bacterial therapies, chest CT showed resolution of the nodule (B, black arrow) and pneumonia.

## Slide 10
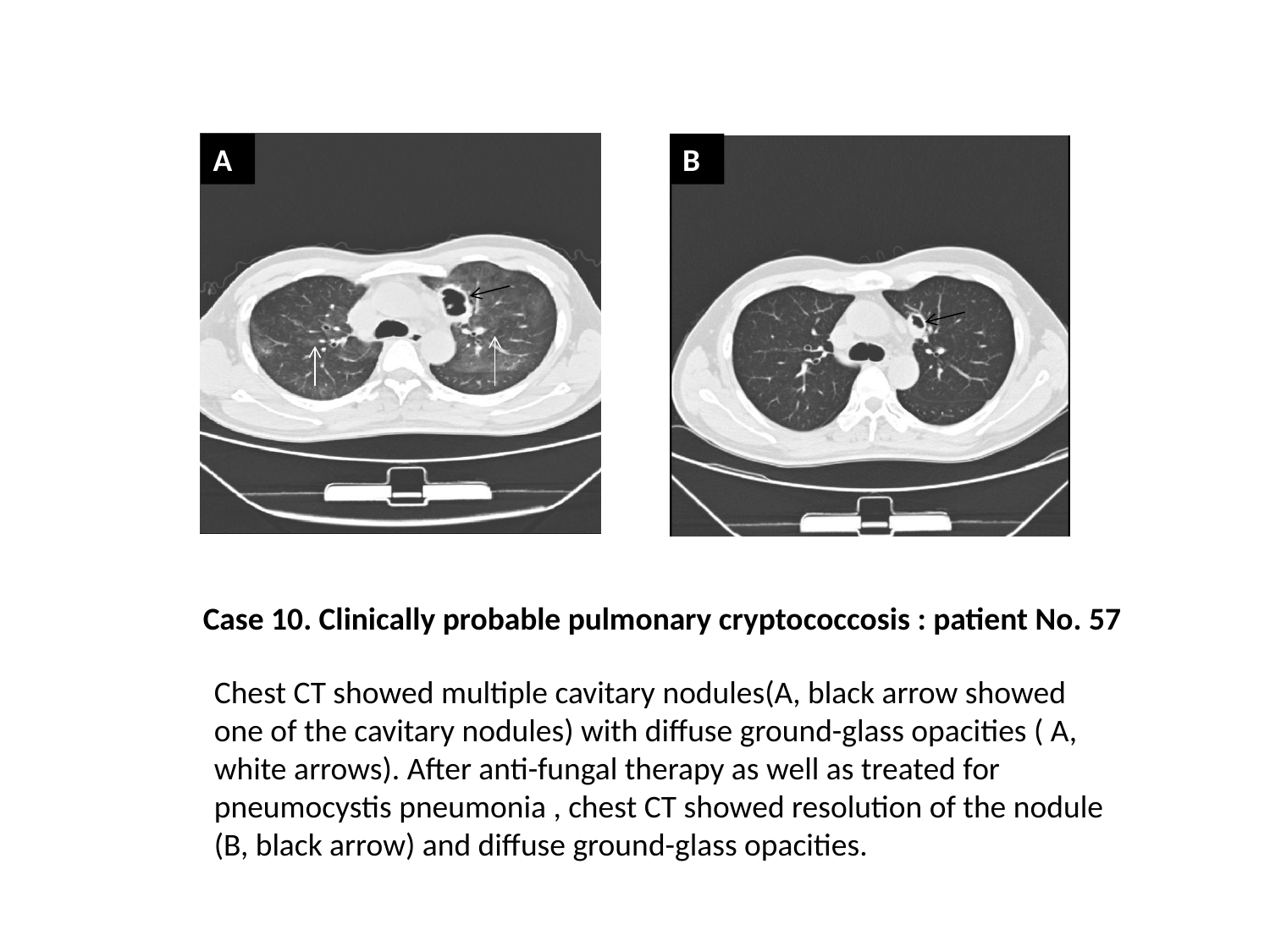

A
B
Case 10. Clinically probable pulmonary cryptococcosis : patient No. 57
Chest CT showed multiple cavitary nodules(A, black arrow showed one of the cavitary nodules) with diffuse ground-glass opacities ( A, white arrows). After anti-fungal therapy as well as treated for pneumocystis pneumonia , chest CT showed resolution of the nodule (B, black arrow) and diffuse ground-glass opacities.

## Slide 11
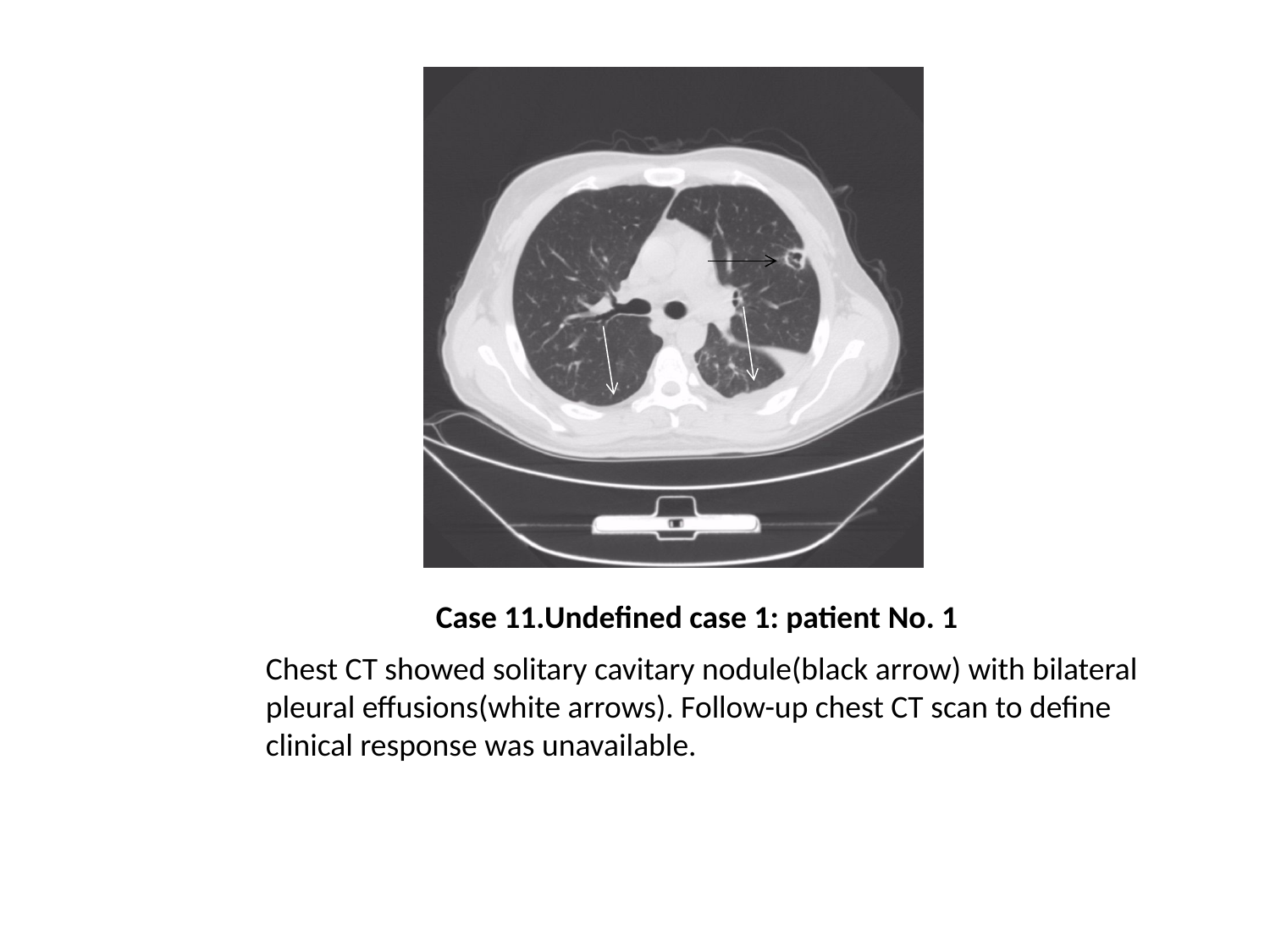

Case 11.Undefined case 1: patient No. 1
Chest CT showed solitary cavitary nodule(black arrow) with bilateral pleural effusions(white arrows). Follow-up chest CT scan to define clinical response was unavailable.

## Slide 12
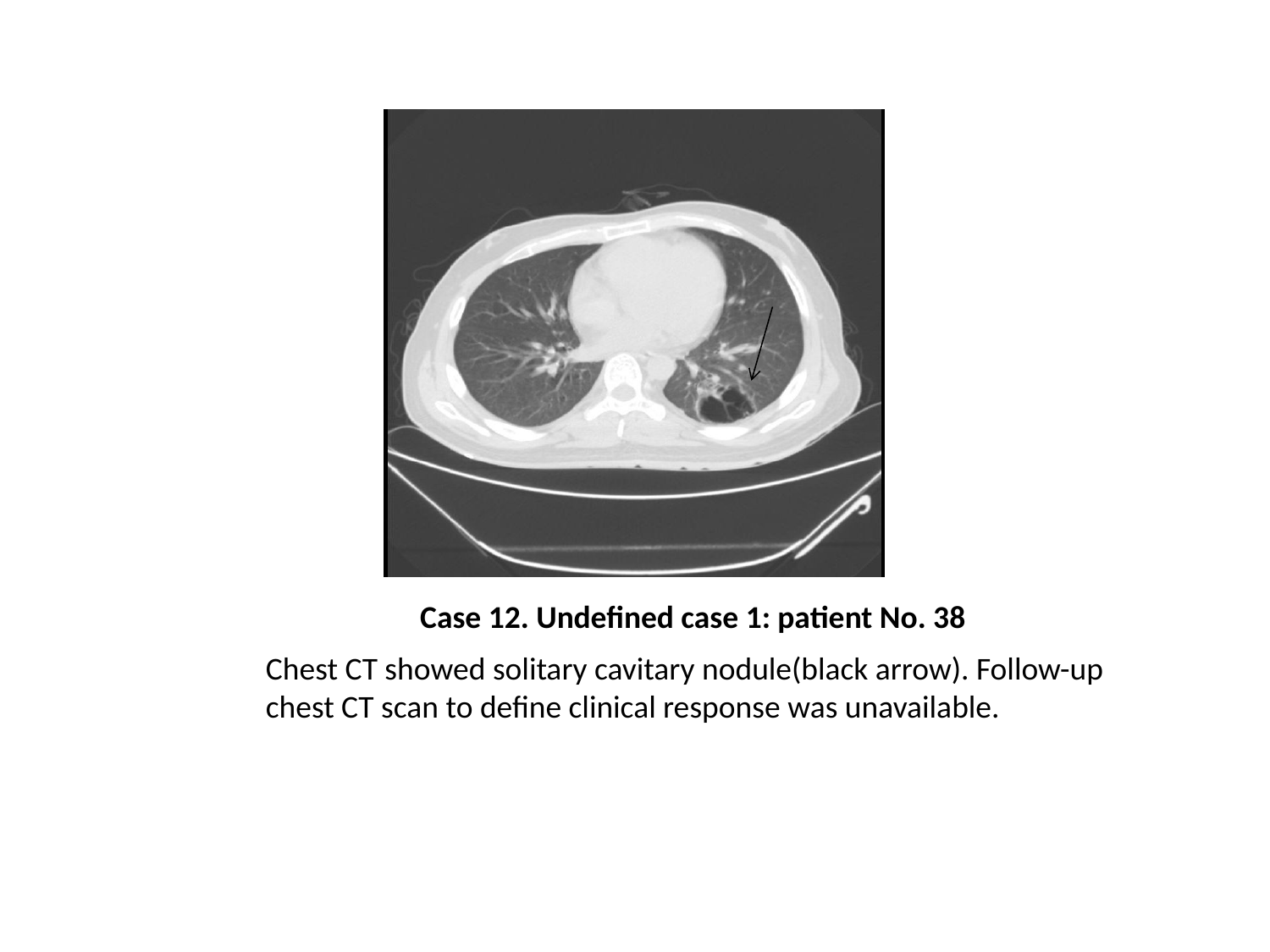

Case 12. Undefined case 1: patient No. 38
Chest CT showed solitary cavitary nodule(black arrow). Follow-up chest CT scan to define clinical response was unavailable.
